# Metabolomic Profile Predicts Development of Microalbuminuria in Individuals with Type 1 Diabetes

**DOI:** 10.1038/s41598-018-32085-y

**Published:** 2018-09-14

**Authors:** Jani K. Haukka, Niina Sandholm, Carol Forsblom, Jeffrey E. Cobb, Per-Henrik Groop, Ele Ferrannini

**Affiliations:** 1 Folkhälsan Institute of Genetics, Folkhälsan Research Center, Helsinki, Finland; 2 Abdominal Center Nephrology, University of Helsinki and Helsinki University Hospital, Helsinki, Finland; 3 Diabetes & Obesity Research Program, Research Program’s Unit, University of Helsinki, Helsinki, Finland; 4 Metabolon, Durham, N.C. USA; 5 Department of Diabetes, Central Clinical School, Monash University, Melbourne, Victoria Australia; 6 CNR Institute of Clinical Physiology, Pisa, Italy

## Abstract

Elevated urinary albumin excretion (microalbuminuria) is an early marker of diabetic nephropathy, but there is an unmet need for better biomarkers that capture the individuals at risk with higher accuracy and earlier than the current markers do. We performed an untargeted metabolomic study to assess baseline differences between individuals with type 1 diabetes who either developed microalbuminuria or remained normoalbuminuric. A total of 102 individuals progressed to microalbuminuria during a median follow-up of 3.2 years, whereas 98 sex-, age- and body mass index (BMI) matched non-progressors remained normoalbuminuric during a median follow-up of 7.1 years. Metabolomic screening identified 1,242 metabolites, out of which 111 differed significantly between progressors and non-progressors after adjustment for age of diabetes onset, baseline glycosylated hemoglobin A1c (HbA_1c_), and albumin excretion rate (AER). The metabolites that predicted development of microalbumiuria included several uremic toxins and carnitine metabolism related molecules. Iterative variable selection indicated erythritol, 3-phenylpropionate, and N-trimethyl-5-aminovalerate as the best set of variables to predict development of microalbuminuria. A metabolomic index based on these metabolites improved the prediction of incident microalbuminuria on top of the clinical variables age of diabetes onset, baseline HbA_1c_ and AER (ROC_AUC_ = 0.842 *vs* 0.797), highlighting their ability to predict early-phase diabetic nephropathy.

## Introduction

Several hundred million people worldwide suffer from diabetes mellitus. While the majority of them have type 2 diabetes, also type 1 diabetes is a growing health problem in the Western world^1^. Roughly one third of individuals with type 1 diabetes develop chronic complications such as diabetic nephropathy (DN), diabetic retinopathy and cardiovascular disease^2^. In particular, the presence of DN is the main cause of end-stage renal disease (ESRD) in many developed countries, and this complication also predisposes to cardiovascular disease. Notably, these complications increase the risk of morbidity and premature mortality manifold^3–5^.

Although there is currently no cure for DN, interventions aimed at blood pressure reduction and improvement in glycemic control have been shown to slow down the progression of the kidney disease^6^. Previous studies have suggested that the slope of the renal function decline is usually linear once the patient has developed progressive kidney disease^7^. Therefore, it would be of utmost importance to identify the individuals at risk early enough to be able to initiate treatment that could prevent or at least retard the decline in renal function and ultimately delay the progression to ESRD.

Albumin excretion rate (AER) and estimated glomerular filtration rate (eGFR) are the most widely used measures to detect DN and to monitor its progression. Attempts to discover novel biomarkers that can identify the disease at an even earlier stage than AER, have been ongoing for many years. However, no other biomarker has been able to convincingly outperform AER thus far^8^.

Using a single biomarker may not be sufficient to detect the subtle underlying pathogenic mechanisms of complex diseases^9^. Biological processes are complex networks of genes, proteins and metabolites, which are subject to tight regulation and feedback loops. Little is known about the metabolic pathways affected in early DN in individuals with type 1 diabetes, especially as previous studies have to a large extent focused on individuals with type 2 diabetes and/or more severe kidney disease. Recent advances in high-throughput metabolomic screening, computational power and methods have opened up new possibilities to investigate whether certain metabolites could better describe the early pathogenic processes leading to progressive kidney failure. Furthermore, if metabolites can be shown to be causative, these metabolites could also be targets for drug intervention.

With the aim of discovering potential novel predictors of DN, we studied the metabolomics profile of individuals who either developed incident microalbuminuria or remained normoalbuminuric during follow up.

## Results

The present study included 200 Finnish individuals with type 1 diabetes with normal AER and an eGFR above 60 mL/min/1.73 m^2^ at the study baseline. We selected 102 individuals (progressors) who had developed microalbuminuria based on their prospective medical data. A total of 98 individuals (non-progressors), who retained normal AER throughout the study, were selected as controls. Both groups had similar sex distribution, age, body mass index (BMI), waist-to-hip ratio (WHR), and eGFR at baseline (Table 1). In the progressors, age at diabetes onset was higher and diabetes duration shorter. In addition, glycemic control was worse, and diastolic blood pressure, serum triacylglycerols and AER were higher in the progressors than in the non-progressors. The progressors developed albuminuria at a median follow-up of 3.2 [inter quartile range (IQR) 3.3] years, and had significantly lower eGFR at the last available visit. In the entire cohort, independent clinical predictors of progression were baseline AER (odds ratio (OR) 2.29 [95% confidence interval (CI) 1.51–3.65]), glycosylated hemoglobin A1c (HbA_1c_) (OR 2.46 [1.65–3.82]), and age of diabetes onset (OR 2.03 [1.40–3.02]). Multivariable logistic regression of these clinical predictors yielded an area under a receiver operating characteristic curve (ROC_AUC_) of 0.797 for progression to microalbuminuria.

**Table 1:** Baseline anthropometric and clinical *parameters.

	Progressors (n = 102)	Non-progressors (n = 98)	*p*
Female/male (%)	51	49	ns
Age (years)	34 ± 12	34 ± 8	ns
BMI (kg/m^2^)	25.3 ± 3.6	24.9 ± 3.0	ns
Waist/hip ratio	0.87 ± 0.08	0.86 ± 0.07	ns
Age of diabetes onset (years)	15 ± 9	11 ± 7	<0.004
Diabetes duration (years)	19 ± 11	23 ± 7	<0.0001
**Baseline**			
HbA_1c_ (%)	9.34 ± 1.65	8.29 ± 1.30	<0.0001
HbA_1c_ (mmol/L)	78,6	68.2	<0.0001
Insulin dose (IU/kg)	0.77 [0.30]	0.74 [0.35]	ns
Systolic BP (mmHg)	130 ± 15	129 ± 12	ns
Diastolic BP (mmHg)	80 ± 10	77 ± 9	<0.05
triacylglycerols (mmol/L)	1.53 ± 1.17	1.12 ± 0.74	<0.001
Total cholesterol (mmol/L)	5.10 ± 1.03	4.81 ± 0.76	ns
HDL cholesterol (mmol/L)	1.31 ± 0.37	1.24 ± 0.33	ns
Serum creatinine (µmol/L)	73 ± 14	72 ± 12	ns
eGFR (mL/min/1.73 m^2^)	108 ± 19	109 ± 13	ns
AER (mg/24 h)	14 [13]	9 [6]	<0.0001
ACEI/AT2RB	12	4	ns
Statins	10	6	ns
**Follow up**			
Follow-up time (years)	6.8 [3.8]	7.6 [4.1]	0.001
Time to progression (years)	3.2 [3.3]	NA	NA
Last eGFR (mL/min/1.73 m^2^)	94 ± 28	105 ± 12	<0.01
Time to last eGFR (years)	10.4 [7.0]	10.6 [6.3]	ns
Change in eGFR (mL/min/1.73 m^2^)	−9 [27]*	−6 [18]*	<0.0001

*Entries are mean ± SD; AER = albumin excretion rate. median [interquartile range]; p values are from c^2^ or Mann Whitney testing; *p < 0.0001 *vs* baseline by Wilcoxon sign-rank test.

### Regression analysis of serum metabolites

Metabolomic screening identified 1,242 peaks, of which 770 were named metabolites and 472 unnamed chemicals. Without adjustment, there were 105 named metabolites for which >90% of the samples had detectable concentrations and for which the difference between the progressors and the non-progressors was statistically significant at *p* ≤ 0.05. In general, many of the carbohydrates were elevated in the progressors, as were most of the fatty acid species, nucleotides, amino acids and their derivatives, and virtually all circulating dipeptides. In contrast, the γ-glutamyl aminoacids (*e*.*g*. γ-glutamylglutamate) were all reduced (Supplementary Table 1). After adjustment for age of diabetes onset, baseline HbA_1c_ and AER, there were 111 metabolites that were nominally significantly (*p* ≤ 0.05) associated with the progression to microalbuminuria and 61 of these metabolites were named ones (Supplementary Table 2). Altogether 10 progressors and 6 non-progressors were using ACEI/AT2RB medication and 12 progressors and 4 non-progressors were using statins at baseline (Table 1). When also adjusted with baseline statin and ACEI/AT2RB usage, 91 metabolites were nominally significantly (*p* ≤ 0.05) associated with the progression to microalbuminuria. In princal component analysis the most progressors and non-progressors were grouped together, supporting that groups were well matched at the baseline in terms of the majority of the metabolites (Supplementary Fig. 1).

### Random Forest analysis of metabolites

**Figure 1:**
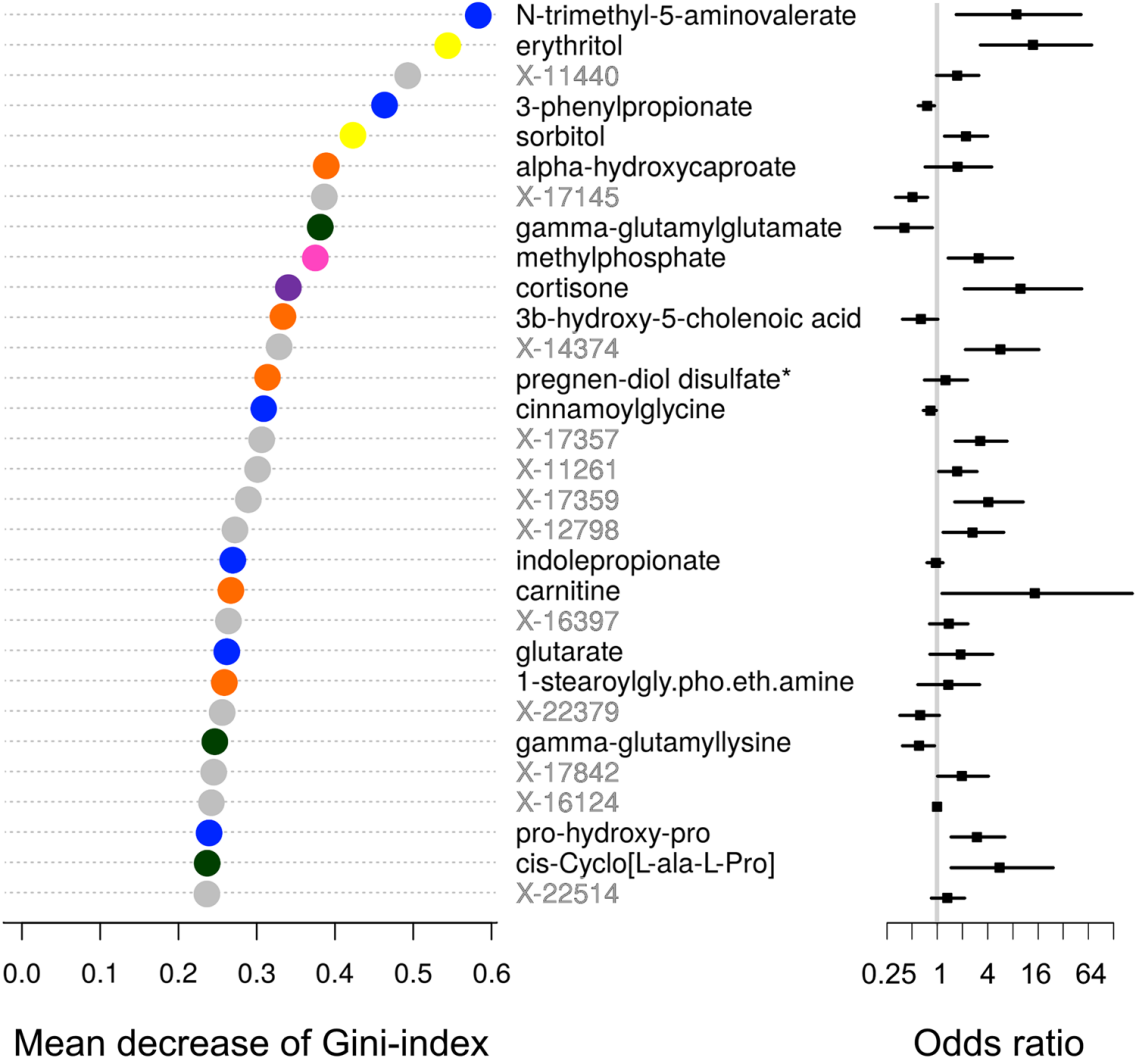
The top 30 metabolites in the RF analysis ordered by Gini-index. Progression to microalbuminuria was set as the response variable and all serum metabolites identified by the platform were set as predictors. Yellow = carbohydrate/polyol, green = peptide intermediate, blue = amino acid intermediate, orange = lipid, violet = nucleotide intermediate, light blue = amino acid, purple = steroid, grey = unknown. The odds ratios for individual metabolites adjusted for age of diabetes onset, baseline HbA_1c_ and AER are shown on the right.

In order to identify the most important metabolites and to exclude associations by chance, we performed a random forest (RF) analysis. The model performance was estimated in samples not included in the training of the corresponding decision trees. The RF model’s out-of-bag error rate was 37.5%. Among the top 30 metabolites identified by RF selection, there were 18 named ones (Fig. 1). These metabolites included carbohydrates (erythritol and sorbitol), nucleotides (methylphosphate), six amino acid and three dipeptide intermediates, and six lipids including the stress hormone cortisone. The highest ranking amino acid intermediate was the N-trimethyl-5-aminovalerate and the top dipeptide intermediates included γ-glutamyl-molecules, of which the γ-glutamylglutamate was the highest ranked one. Within the seven lipids among the top 30 RF metabolites, α-hydrocaprocate was the top ranked one. Of the top 30 metabolites only sorbitol had more than 10% of observations missing (16.5%).

**Figure 2:**
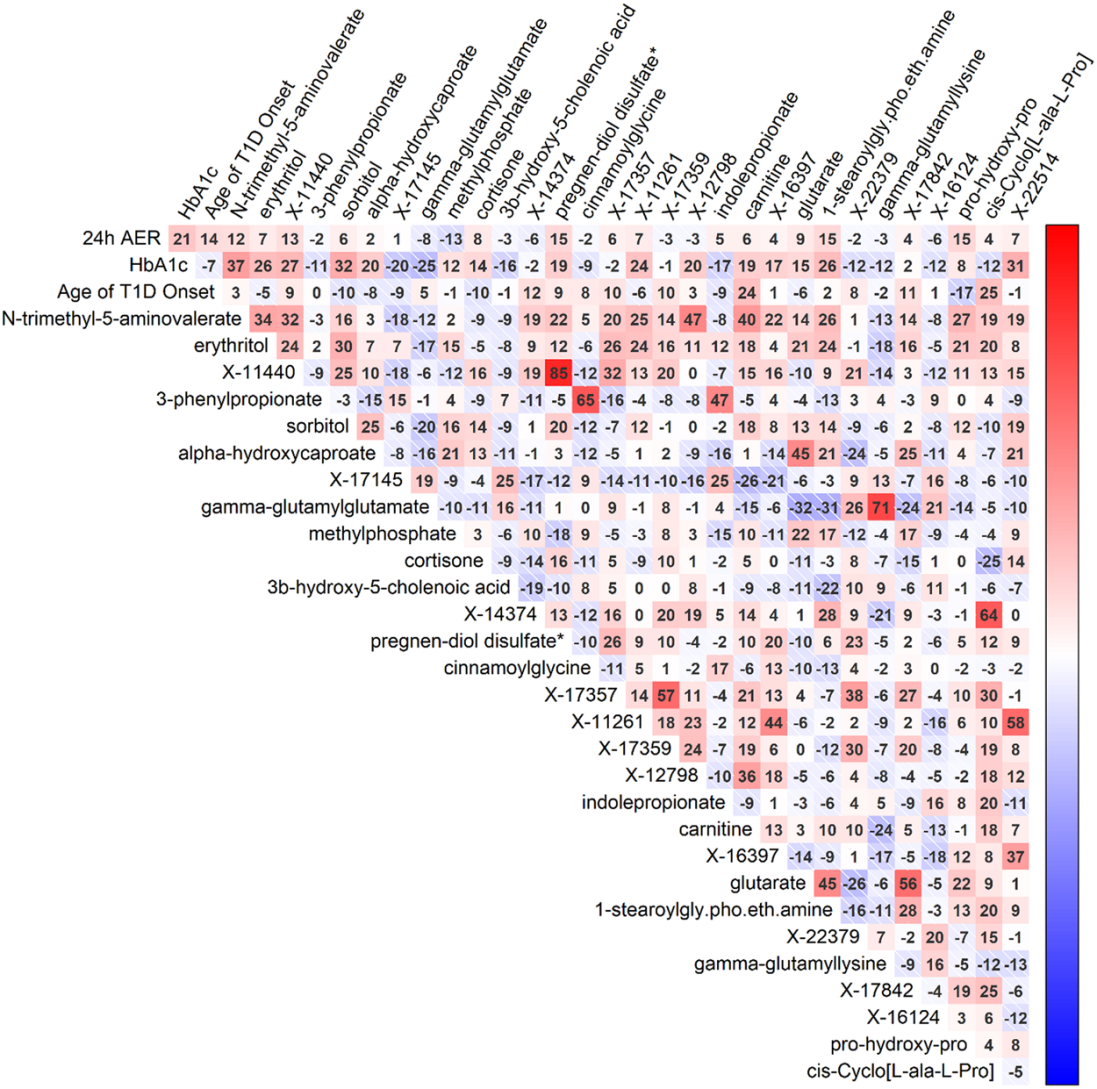
Correlation plot for the top 30 random forest selected metabolites and the three clinical variables age of diabetes onset, baseline HbA_1c_ and AER. The values [−100, 100] represent correlation coefficients which were multiplied by 100. The clinical variables showed only weak to modest correlations with the top 30 RF selected metabolites (highest between HbA_1c_ and N-trimethyl-5-aminovalerate, *r* = 0.37). The strongest correlations between the metabolites can be seen between X-11440 and pregnen-diol disulfate (*r* = 0.85) and between γ-glutamylglutamate and γ-glutamyllysine (*r* = 0.71).

The top 30 RF-selected metabolites showed weak or modest correlations with age of diabetes onset, baseline HbA_1c_ and AER (Fig. 2). Notably, HbA_1c_ correlated modestly with N-trimethyl-5-aminovalerate (*r* = 0.37) and sorbitol (*r* = 0.32). The strongest correlations between the metabolites were seen between X-11440 and pregnen-diol disulfate (*r* = 0.85) as well as between the γ-glutamylglutamate and γ-glutamyllysine (*r* = 0.71).

### Variant selection of Random forest analysis

To find a small set of variables with good prediction performance, we performed variable selection refinement with Variable Selection Using Random Forests software (VSURF) including all metabolites. The run was repeated ten times in order to assess the robustness of the selection. The result showed that three metabolites X-21365 (N-trimethyl-5-aminovalerate; personal communication from Metabolon), erythritol and 3-phenylpropionate were included in the final predictive model in all ten runs and one additional metabolite, γ-glutamyllysine, in one run (Supplementary Fig. 2). The out-of-bag errors for these 10 VSURF models were 0.25–0.27. All these metabolites were also significant in the univariate logistic regression analysis after adjustment for the clinical factors (Supplementary Fig. 3).

As a sensitivity test, we performed another ten-fold VSURF run for all metabolites except the three metabolites selected by the VSURF in the first 10 runs. The resulting models needed 8–12 metabolites to reach similar out-of-bag error as the models that consisted of N-trimethyl-5-aminovalerate, erythritol and 3-phenylpropionate (Supplementary Fig. 2). There was also much greater dispersion of the metabolites included in these models as altogether 37 metabolites were selected, suggesting that the three originally selected ones are essential for predicting microalbuminuria. The three original VSURF-selected metabolites showed modest to strong correlations with the 30 other RF-selected metabolites (*i*.*e*. 3-phenylpropionate – cinnamoylglycine *r* = 0.65, N-trimethyl-5-aminovalerate – carnitine *r* = 0.40, erythritol – sorbitol *r* = 0.30) (Fig. 2).

### Metabolic index

**Figure 3:**
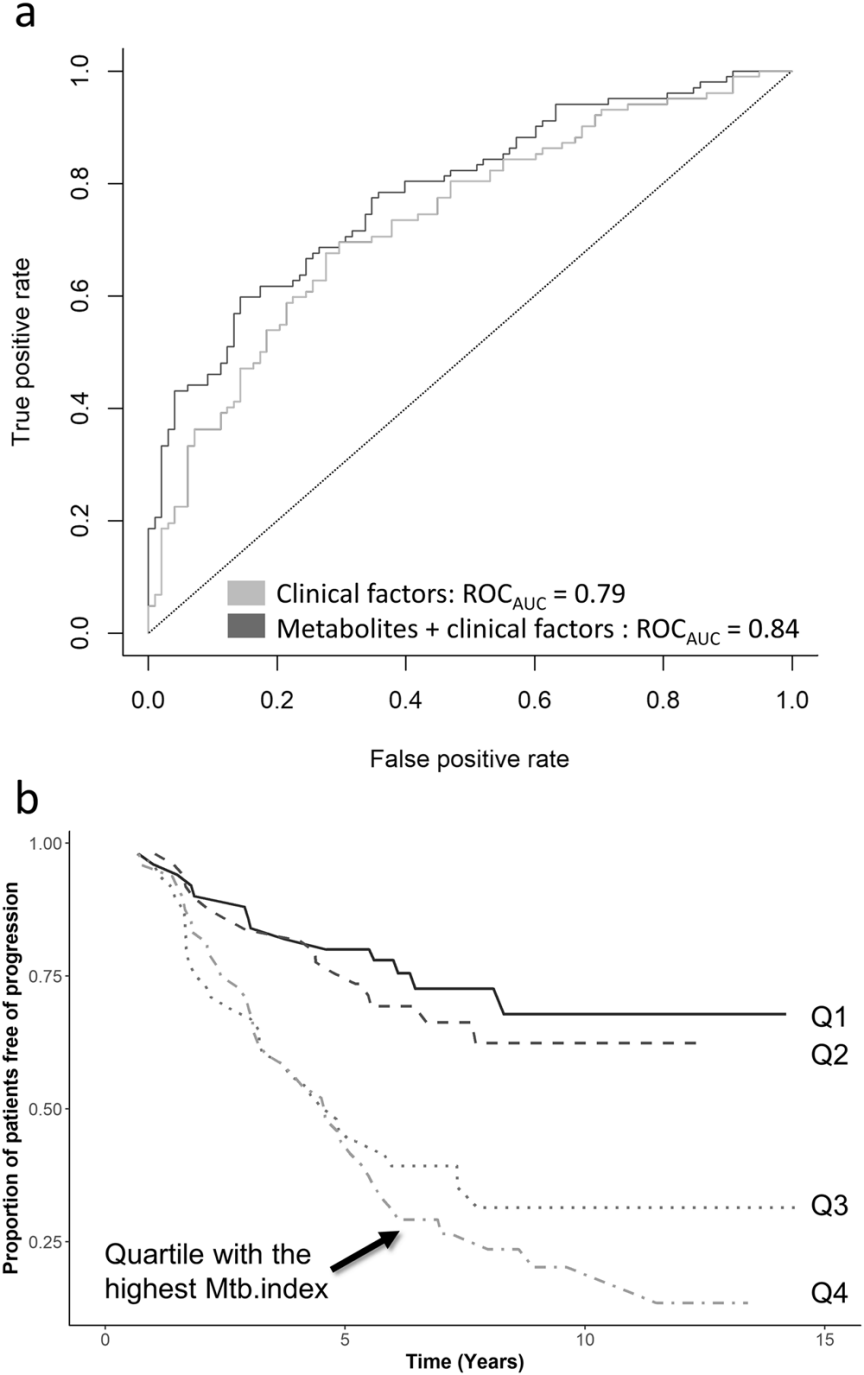
(**a**) When the Mtb.index is added to the most significant clinical factors (age of diabetes onset, baseline HbA_1c_ and AER), ROC_AUC_ increases to 0.842 compared to 0.797 for clinical variables only. (**b**) Survival plot of progression to microalbuminuria of individuals with type 1 diabetes by quartiles of metabolomics index (Mtb.index). Individuals in the two top quartiles (i.e. Mtb.index above median) showed more rapid progresion to the microalbuminuria compared to the patients at the bottom quartiles.

To study the combined predictive value of the three VSURF-selected metabolites, we formed a metabolomic index (Mtb.index) based on linear combination of the three metabolites N-trimethyl-5-aminovalerate, erythritol, and 3-phenylpropionate. This Mtb.index was strongly associated with the progression to microalbuminuria with an OR of 2.96 (*p* = 2.75 × 10^−7^). The ROC_AUC_ based on the Mtb.index alone was 0.736. Importantly, the Mtb.index improved the predictive performance when added on top of the most predictive clinical variables AER, age of diabetes onset and HbA_1c_. Consequently, the ROC_AUC_ improved significantly from 0.797 for clinical variables only, to ROC_AUC_ = 0.842, for the clinical variables plus Mtb.index (DeLong test’s *p-*value = 0.017) (Fig. 3a). Furthermore, the three metabolites improved the prediction of incident microalbuminuria when added on top of the clinical model including the long-term mean HbA_1c_ (median 6 [IQR:1.35–10.65] measurements per patient by the time of the baseline visit) instead of the baseline HbA_1c_ (ROC_AUC_ 0.82 vs. 0.72) (Supplementary Fig. 4).

When individuals in the top quartile of the Mtb.index were compared with the rest of the cohort, the survival plot showed that the individuals in the two top quartiles had a more rapid progression to microalbuminuria compared to the individuals at the bottom quartiles (Fig. 3b).

## Discussion

In the present study, we assessed baseline metabolomic differences between individuals with type 1 diabetes, who later developed incident microalbuminuria, and those for whom AER remained normal throughout the follow-up. First, we observed that the metabolomic profile of progressors and non-progressors differed significantly with respect to several metabolites; a logistic regression analysis resulted in 111 nominally significant metabolites after adjustment for age of diabetes onset, baseline HbA_1c_ and AER. Second, the metabolites as highlighted by RF included molecules from several biochemical groups such as polyols, amino acid-, peptide- and nucleotide intermediates, lipids and the stress hormone cortisone (Fig. 1). Notably, among the most important metabolites there were also several ones with unknown chemical structure. Ultimately, with the aim to identify a minimal set of important metabolites, three variables were selected for a prediction model with the VSURF method: namely, two known ones, erythritol and 3-phenylpropionate, as well as an unknown one, X-21365. This unknown metabolite has retention index of 973 and molecular mass of 160.133 Da and it has recently been identified as N-trimethyl-5-aminovalerate (Metabolon, personal communication).

The three metabolites verified by VSURF, as well as several other of the top metabolites selected by RF, have previously been highlighted in studies of factors associated with DN such as weight gain, insulin resistance, and gut microbiota activity. N-trimethyl-5-aminovalerate (formerly known as trimethyl-N-aminovalerate, 5-trimethylaminovalerate, Nδ-trimethyl-5-aminopentanoate, 5-N-trimethylaminopentanoate, γ-butyrobetaine[GBB]-5 or X-21365 on the Metabolon platform) was the first variable selected by VSURF and the metabolite with the highest importance in RF. It was significantly elevated in the progressors. Interestingly, a recent study found that, together with citrulline, it was the only serum metabolite that was significantly elevated in metformin treated type 2 diabetic patients when compared to non-treated patients^10^. N-trimethyl-5-aminovalerate was also found to be the most significant metabolite associated with low-fat milk intake in two recent studies^11,12^. The exact production pathway for the metabolite is currently unknown but N-trimethyl-5-aminovalerate is likely methylated from 5-aminovalerate^11^. 5-aminovalerate is a degradation product of lysine or proline by gut microbiota^11,13^; it has also been linked to the carnitine metabolism. In our study it was moderately correlated with carnitine (*r* = 0.40). Carnitine was significant in the logistic regression and the 20^th^ most important metabolite in the RF selection.

The second metabolite selected by VSURF, erythritol, is a polyol that has been used as a low-calorie sweetener. Although erythritol has been thought to be a xenobiotic, *i*.*e*. a metabolite that is not synthetized or metabolized in the human body, a recent study suggests that erythritol is synthesized from glucose in the pentose-phosphate pathway and then metabolized to erythronate^14^. Recently, elevated erythritol concentrations have been found in individuals with diabetic retinopathy, cardiovascular events, and weight gain in young adults^10,15,16^. One of the main hypotheses is that increased polyol pathway flux is responsible for vascular complications in diabetes^17^. Furthermore, elevated erythritol levels have been observed in individuals with transaldolase deficiency^18^. In addition to erythritol, another carbohydrate sorbitol was also significantly elevated in the progressors, and was also one of the most important metabolites selected by RF (Fig. 1).

3-phenylpropionate was the third metabolite selected by VSURF. 3-phenylpropionate was among the top metabolites for insulin sensitivity in a non-diabetic population in an earlier RF based study^19^. It is metabolized by gut microbiota such as *E*. *coli*, which can process it to 2-hydroxypenta-2,4-dieneoate and succinate, which go further into the toluene pathway and the tricarboxylic-acid cycle^20^. In addition to 3-phenylpropionate, many of the other RF selected metabolites, including cis-Cyclo[L-ala-L-Pro] and γ-glutamylglutamate are amino acid and peptide intermediates, which suggests that amino acid metabolism also plays a role in the progression to microalbuminuria.

Only a few studies have assessed the metabolomic profile for early-stage renal complications in individuals with type 1 diabetes. Mäkinen *et al*. used NMR screening of 50 serum metabolites and the self-organizing map method to classify individuals with type 1 diabetes, who either developed microalbuminuria or remained normoalbuminuric, and discovered both protecting and predisposing metabolomic profiles^21^. Using similar patient grouping, van der Kloet *et al*. studied metabolomics differences in the urine with 130 GC-MS and 89 LC-MS identified metabolites^22^. They found that acyl-carnitine-, acyl-glycine- and tryptophan-metabolism related compounds showed the most significant difference between the patient groups. However, these earlier studies included a smaller amount of metabolites and employed different analysis methods or medium, making it difficult to compare them with the current study.

A modern high-throughput metabolomics platform, such as that used in the present study, is capable of discriminating more than a thousand metabolites per serum sample. The same platform was employed in our previous study in individuals with type 2 diabetes. That study used RF analysis to investigate the metabolomic differences in serum and urine between individuals who developed microalbuminuria and/or eGFR declined below < 60 mL/min/1.73 m^2^ and those in whom AER and eGFR remained normal^23^. Importantly, the serum metabolomic profile from the current cohort of individuals with type 1 diabetes that progressed to microalbuminuria differed substantially from the top metabolites of our previous type 2 diabetes study. For example, the RF classifier in the previous type 2 diabetes study resulted in three 1-acylglycerol molecules among the top 5 significant metabolites. The individuals in the type 2 diabetes study were however considerably older when compared to the participants in the type 1 diabetes study. These differences in predictors of progression between individuals with type 2 and type 1 diabetes could be due, at least in part, to the fact that diabetic kidney disease in individuals with type 2 diabetes is more heterogeneous than in type 1 diabetes. In the latter, diabetic kidney disease is nearly always DN, while in type 2 diabetes some individuals may have true DN but the majority may have diabetic kidney disease due to other etiologies such as hypertension, obesity and aging.

Using the same platform, Niewczas *et al*. studied metabolomic differences between individuals with type 2 diabetes, who progressed to ESRD, and those whose kidney function remained stable, and found increased concentrations of uremic metabolites in the progressors^24^. Interestingly, among the RF selected metabolites in our study, there were also uremic solutes, including erythritol and the amino acid intermediates indolepropionate and cinnamoylglycine. This could indicate that these metabolites play a role in the disease progression both in early- and late stage DN.

The uremic toxin C-glycosyltryptophan has previously been associated with declining eGFR in chronic kidney disease in individuals with type 2 diabetes and also with the progression to ESRD in individuals with type 1 and type 2 diabetes^23–25^. Although the C-glycosyltryptophan concentrations were significantly elevated among the progressors, the RF classification did not select C-glycosyltryptophan among the most significant metabolites for the early DN.

Previous studies have shown that in comparison with other machine learning based classifiers such as projection to latent structures (PLS), support vector machine (SVM), and linear discriminant analysis (LDA), the RF is a robust classifier for metabolomics studies^26^. For example, biomarker panels consisting of 3–5 top metabolites and proteins selected by the RF showed superior performance compared to the currently used single marker Prostate Specific Antigen in predicting the progression of prostate cancer at different stages^27^.

The current study is limited by its relatively small number of samples. However, the progresssors and the non-progressors had similar distribution in sex, age, BMI, and waist to hip ratio, and the sample size matches or exceeds the size of similar previous metabolomic studies for other diseases. Another limitation is that we did not have a replication cohort in which the performance of the Mtb.index could be evaluated. However, as discussed earlier, random forest subsets the dataset during the algorithm run, which diminishes the need for arbitrary division of the dataset. The VSURF-selected metabolites erythritol, 3-phenylpropionate and N-trimethyl-5-aminovalerate were also consistently ranked among the highest in the RF and they were also highly significant in the logistic regression. However, in order to generalize the findings, more research on non-Finnish populations is needed.

The −20 °C storage temperature may also have affected the stability and concentrations of certain metabolites, and thus we may have missed some important metabolites. Nevertheless, the storage conditions were similar for the case and control samples, and therefore this should not result in false positive findings.

It is also worth highlighting that over one third of all metabolites (and 12 of the top 30) selected by RF were unknown. For example, X-11440, the third most important metabolite selected by the RF has been associated with serum urate regulation^28^. Therefore, there is an urgent need for better metabolite identification in order to get a more complete picture of disease prediction.

This study has identified a set of metabolites that improves prediction of incident microalbuminuria in individuals with type 1 diabetes beyond commonly used clinical variables and AER. However, we did not assess whether these metabolites also predict the risk of progression from microalbuminuria to macroalbuminuria or even to more severe DN with reduced kidney function. This will be an important next step in metabolomic research, since there might be different factors involved in the initiation of DN than in the progression of already established DN. Nevertheless, based on this study, measuring erythritol, 3-phenylpropionate, and N-trimethyl-5-aminovalerate could be a useful tool to detect individuals at risk already at an earlier stage. Further research is needed to explore if these metabolites are involved in the pathogenesis of DN in a causative manner, and if they could serve as potential targets for intervention.

## Methods

### Patients

This study is part of the ongoing nationwide, multi-center, prospective Finnish Diabetic Nephropathy Study (FinnDiane) that aims to identify risk factors for diabetic complications with particular emphasis on DN. More than 5,000 individuals with type 1 diabetes have thus far been recruited into the FinnDiane study at their regular visits to the attending physician. Type 1 diabetes was defined as age at onset of diabetes below 40 years and insulin treatment initiated within one year of diagnosis. Data on recruitment and clinical characterization of the participants have been presented in detail elsewhere^29^. The study protocol was approved by the Ethical comittee of the Helsinki and Uusimaa Hospital District as well as by the local ethics committees at each FinnDiane participating center, and all patients signed a written informed consent. The study was performed in accordance with the Declaration of Helsinki.

All participants were followed either through a prospective visit carried out in the same manner as the baseline visit or alternatively in a small number of individuals by assessing the medical records and all available routine laboratory data, if the patient had not yet participated in a scheduled follow-up visit. At these baseline and follow-up visits, the participants underwent a thorough clinical examination and completed a standardized questionnaire regarding history of complications, medication, family history, and lifestyle. Serum and urine samples were collected for the determination of biochemical variables such as creatinine, lipids, HbA_1c_, and urinary AER^29^.

For the present study, we identified individuals, who at baseline had normal AER and an eGFR above 60 mL/min/1.73 m^2^, and for whom follow-up data on kidney status were available. We then selected 102 consecutive individuals, who developed microalbuminuria during a median follow-up of 3.2 years (progressors), and matched them for sex, age, and BMI with 98 individuals, who remained normoalbuminuric during a median follow-up of 7.1 years (non-progressors). The clinical characteristics of the two groups are given in Table 1.

The AER value used in the analyses was obtained from a 24-hour urine collection in the absence of symptoms and signs of urinary tract infection or other interfering clinical conditions. The urinary albumin concentration of this sample was measured centrally by an immunoturbidimetric method. However, the classification of the participants was based on all available data on AER determined locally. Thus, normal AER was defined as AER < 30 mg/24 h (or < 20 µg/min) and microalbuminuria as AER ≥ 30 mg/24 h in at least two out of three overnight or 24 h urine collections. For the metabolomics analysis, a serum sample was taken at baseline between 1998 and 2006 and labeled with a blinded identification code, which was used to track sample handling, results, and data analysis. Blood samples were drawn in the morning after a light breakfast, and the participants were asked to avoid smoking and coffee intake in the morning before the sampling. The aliquots were frozen within 4 hours after the sampling and were stored at −20 °C until processing. The median storage time before metabolomics analysis was 14 years.

Serum creatinine was determined centrally with Jaffé’s method until January 7, 2002 and thereafter by an enzymatic method. Based on duplicate measurements of serum creatinine with the two different methods, all values were transformed to an IDMS traceable value before estimation of the glomerular filtration rate (eGFR) with the CKD-EPI equation^30^. Serum lipid (triacylglycerols, cholesterol, HDL-cholesterol) concentrations were also analyzed centrally by automated enzymatic methods (Hoffmann-LaRoche, Basel, Switzerland). HbA_1c_ was determined locally with standard methods.

### Metabolomics

Non-targeted metabolomics profiling was performed by Metabolon Inc., as previously described and in Supplementary Methods^23,31–33^. In brief, samples were prepared using an automated MicroLab STAR® system (Hamilton Co). Sample preparation included an aqueous methanol extraction process in order to remove the protein fraction while allowing maximum recovery of small molecules. The resulting extract was sent for global untargeted metabolomics analysis by Ultrahigh performance liquid chromatography/Mass Spectroscopy (UPLC/MS/MS) (positive and negative modes) and gas chromatography/Mass Spectroscopy (GC/MS).

*UPLC/MS/MS*. The UPLC/MS/MS platform was based on a Waters ACQUITY ultra-performance liquid chromatography and a Thermo-Finnigan linear trap quadrupole mass spectrometer, which consisted of an electrospray ionization source and linear ion-trap mass analyzer. All extracts were gradient eluted using water and methanol. In addition, 0.1% formic acid was added to extracts reconstituted in acidic conditions, whereas the basic contained 6.5 mM Ammonium Bicarbonate.

*GC/MS*. The samples destined for GC/MS analysis were re-dried under vacuum desiccation for a minimum of 24 hours prior to being derivatized under dried nitrogen using bistrimethyl-silyl-triflouroacetamide (BSTFA). GC column was 5% phenyl and the temperature ramp was from 40 °C to 300 °C in a 16-min period. Samples were analyzed on a Thermo-Finnigan Trace DSQ fast-scanning single-quadrupole mass spectrometer using electron impact ionization.

#### Compound Identification

Raw data were extracted, peak-identified and QC processed using Metabolon’s hardware and software. Compounds were identified by comparison to library entries on retention time/index (RI), mass to charge ratio (*m/z)*, and chromatographic data (including MS/MS spectral data) for purified standards or recurrent unknown entities. The naming of the metabolites was done according to Metabolon’s standards.

#### Data pre-processing

Metabolite concentrations are expressed as relative intensities. Missing values (if any) were imputed to the lowest measured value and metabolite data were scaled proportionately to a median of 1.

### Statistical analysis

Values are expressed as mean ± standard deviation (SD). Because of its skewed distribution, AER is expressed as median [IQR] and was log-transformed for use in the multivariable analyses. Group comparisons of clinical variables were carried out with the χ^2^ or Mann-Whitney test; group differences for metabolites were tested by the Welch test for unequal variances. Multivariable logistic analysis was performed using the forward elimination method. Results are given as OR with 95%CI (calculated per SD of predictor) along with the *c* statistic (*i*.*e*. ROC_AUC_). Principal component analysis was conducted using prcomp method from R stats library. A significance level of *p ≤ *0.05 was utilized in all tests. R (v. 3.3.1) and SPSS (v. 24, IBM, USA) were used for the analyses.

#### Random Forest analysis

The data were elaborated by using RF analysis^34^. This method employs a multistage decision process that attempts to identify and rank relationships between the predictive variables and the response variable. The RF algorithm is an extension of the decision trees method. In short, the algorithm involves generation of a large amount of decision trees, and elements are classified by taking a majority vote among the trees. In each tree, random subsets (N = square root of total number) of variables are selected. Furthermore, each tree is grown using a particular bootstrap sampling (random sampling with replacement). As about one third of the samples left out from each tree is used as test set to estimate the tree’s out-of-the-bag error, there is no explicit need for arbitrary separation into training and test sets. We performed the RF classification by using the RF R-package v. 4.6–12^35^. All serum metabolites were set as predictive variables and the progression to microalbuminuria was set as the response variable. We set the method to generate 20,001 trees for a RF in order to improve the robustness of the classifier. The mean decrease in classification accuracy and the Gini score were used to measure variable importance.

#### Two-step Random Forest Variable Selection

Due to the high amount of metabolites compared to the number of samples, we further validated the selection of the most important variables with iterative random forest variable selection refining implemented in the VSURF R-package^36^. When the method is set to aim for small number of model predictors, it first generates several random forests, and drops the least important variables from the model based on the mean variable importance between the runs. In the second forward selection step, the variables are iteratively added to the model starting from the one with the highest importance. A given variable is added to the final model only if its addition results in a significant decrease in the out-of-the-bag error in the samples not included in the corresponding decision tree in the new RF.

## Electronic supplementary material

Supplementary Material

## Data Availability

The datasets generated and/or analyzed during the current study are not publicly available as the patients’ written consent does not allow data sharing. Data are locally available from the corresponding author on reasonable request.
