# Supplementary material for: Metabolomic Profile Predicts Development of Microalbuminuria in Individuals with Type 1 Diabetes
Source: Sci Rep. 2018 Sep 14;8:13853. doi: 10.1038/s41598-018-32085-y (PMC6138633; doi:10.1038/s41598-018-32085-y)
Supplement: Supplementary file 1 — Supplementary Material [file 41598_2018_32085_MOESM1_ESM.docx]

**ELECTRONIC SUPPLEMENTARY MATERIAL**

**Metabolomic Profile Predicts Development of Microalbuminuria in Individuals with Type 1 Diabetes**

**Jani K Haukka,^1,2,3^, Niina Sandholm,^1,2,3^, Carol Forsblom,^1,2,3^, Jeffrey E. Cobb,^4^, Per-Henrik Groop,^1,2,3,5^, Ele Ferrannini,^6^**

**^1^ Folkhälsan Institute of Genetics, Folkhälsan Research Center, Helsinki
^2^ Abdominal Center Nephrology, University of Helsinki and Helsinki University Hospital, Helsinki, Finland
^3^ Diabetes & Obesity Research Program, Research Program’s Unit, University of Helsinki, Finland**

**^4^ Metabolon, Durham N.C., U.S.A
^5^ Department of Diabetes, Central Clinical School, Monash University, Melbourne, Victoria, Australia;  ^6^ CNR Institute of Clinical Physiology, Pisa, Italy**

**Table of contents**

Contents

[Supplementary Metohds 2](#_Toc519493581)

[Supplementary Figure 1 — Principal component analysis (PCA) 3](#_Toc519493582)

[Supplementary Figure 2 — Three metabolites are sufficient to robustly predict the development of microalbuminuria. 4](#_Toc519493583)

[Supplementary Figure 3 — Individual odds ratios for metabolites that were selected by VSURF 5](#_Toc519493584)

[Supplementary Figure 4 — ROC-curve 6](#_Toc519493585)

[Supplementary Table 1 – Metabolomic profile of selected metabolites 7](#_Toc519493586)

[Supplementary Table 2 – 111 metabolites with nominally significant P-value after adjustment for clinical factors 8](#_Toc519493587)

[Supplementary Table 3 – The Finnish Diabetic Nephropathy Study Centers 12](#_Toc519493588)

# Supplementary Metohds

**QA/QC:** To improve QA/QC, extra samples were included for analyses every day. The samples were taken from a pool of well-studied and characterized human sera. Test samples were randomly distributed during the run, and QC samples were equally spaced between them. A selection QC compounds was carefully chosen not to interfere with the measurement of the test compounds, and added to every sample for chromatographic alignment.  Instrument variability was determined by calculating the median relative standard deviation (RSD) for the standards that were added to each sample prior to injection into the mass spectrometers.  Overall process variability was determined by calculating the median RSD for all endogenous metabolites (i.e., non-instrument standards) present in 100% of the pooled matrix samples.  Experimental samples were randomized across the platform run and QC were samples spaced evenly among the injections.

**Data extraction and compound identification**

Raw data were extracted, peak-identified, and QC-processed by Metabolon, using their hardware and software. Metabolon maintains a library based on authenticated standards that contains the retention time/index (RI), mass to charge ratio (m/z), and chromatographic data (including MS/MS spectral data) on all molecules present in the library. Moreover, biochemical identifications are based on three criteria: retention index within a narrow RI window of the proposed identification, nominal mass match to the library ± 0.2 amu, and the MS/MS forward and reverse scores between the experimental data and authentic standards. The MS/MS scores are based on a comparison of the ions present in the experimental spectrum to the ions present in the library spectrum. Although molecules may share similarities based on one of these factors, the use of all three data points can be utilized to distinguish and differentiate biochemicals. Identification of known chemical entities was based on comparison to library entries of purified standards. Additional mass spectral entries have been created for structurally unnamed biochemicals, which have been identified by virtue of their recurrent nature (both chromatographic and mass spectral). These compounds have the potential to be identified by future acquisition of a matching purified standard or by classical structural analysis.

# Supplementary Figure 1 — Principal component analysis (PCA)

In regards to two first principal components, the progressors and non-progressors were not separated into clear groups but were grouped together, supporting that the progressors and nonprogressors were well matched at the baseline across the majority of the metabolites.


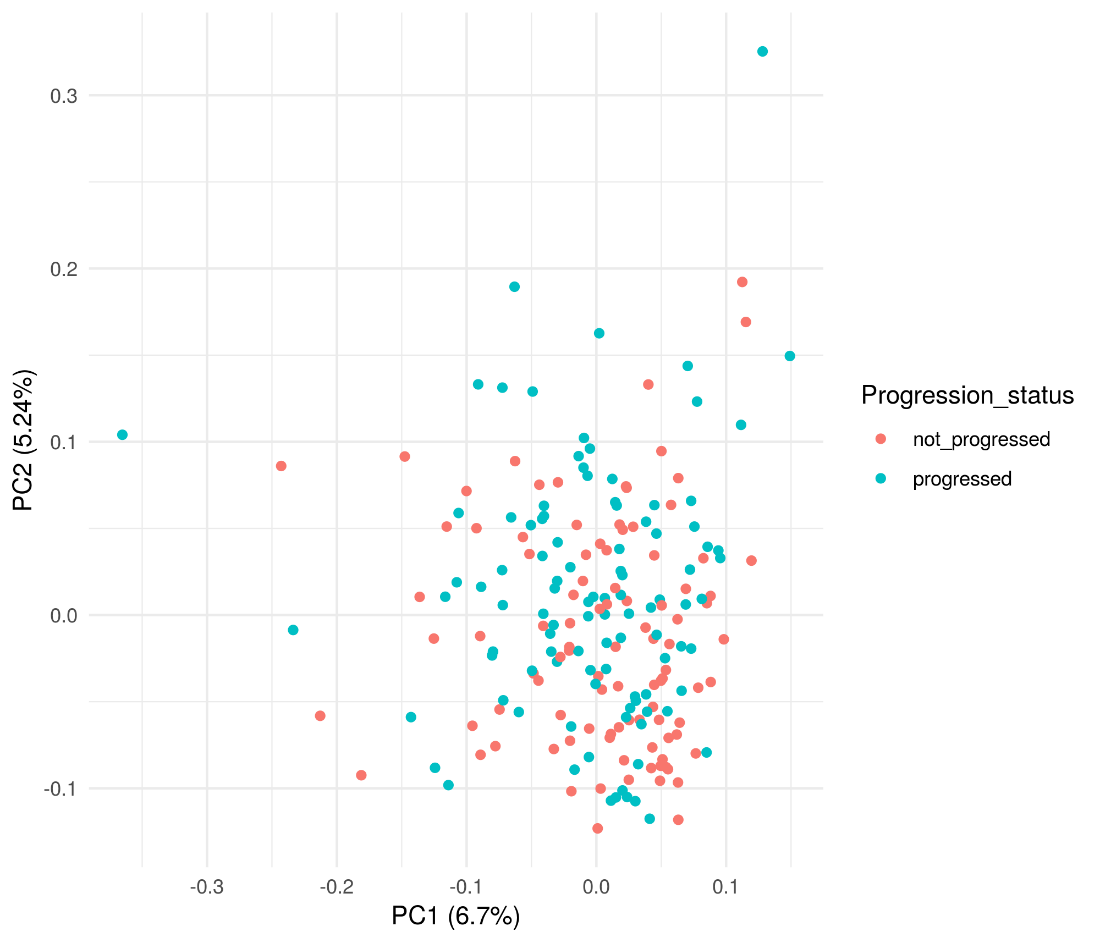


Supplementary Figure 2 — Three metabolites are sufficient to robustly predict the development of microalbuminuria. To find a small set of metabolites with good prediction performance for microalbuminuria we performed 10x VSURF runs. Model fit is evaluated by its out-of-bag (OOB) errors. When VSURF was run with all metabolites (solid line), VSURF selected three metabolites, N-trimethyl-5-aminovalerate, erythritol and 3-phenylpropionate into the model in each of the 10 runs and, in addition to them, γ-glutamyllysine on 1/10 runs. Secondly, VSURF was rerun for 10 times for all metabolites except the three metabolites selected by the VSURF in the first 10 runs (dotted line). The model errors for first 10 VSURF models (with only 3-4 metabolites) settled to 0.25 – 0.27. For the other 10 models without these 3 metabolites, the models needed to include 8-12 metabolites to reach similar performance. There was also much greater dispersion in the models without the top 3 metabolites, as altogehter 37 metabolites were selected into the models by VSURF during 10 runs.


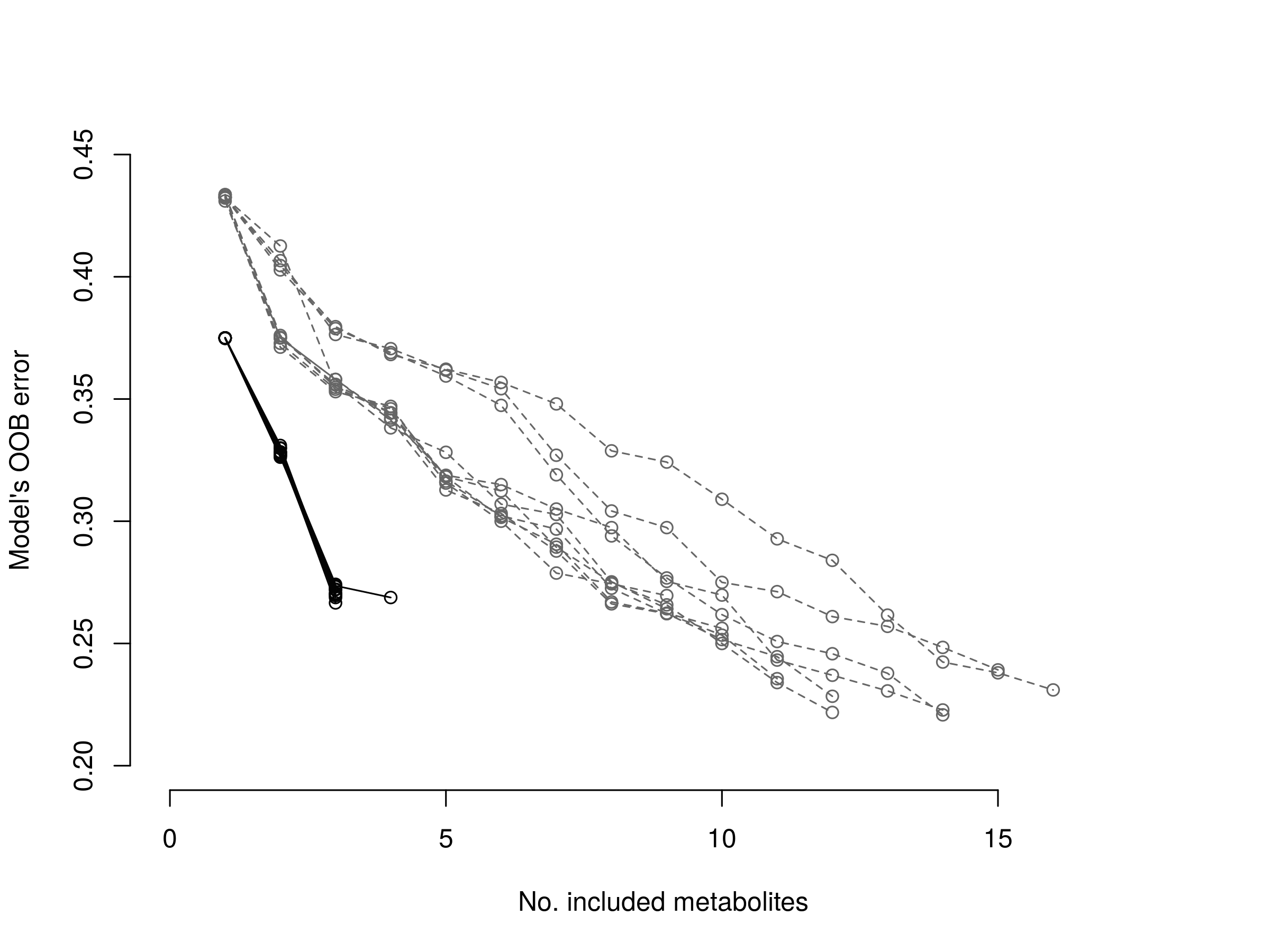


Supplementary Figure 3 — Individual odds ratios for metabolites that were selected by VSURF**. The odds ratios are adjusted with age of diabetes onset, baseline HbA_1c_ and AER.**


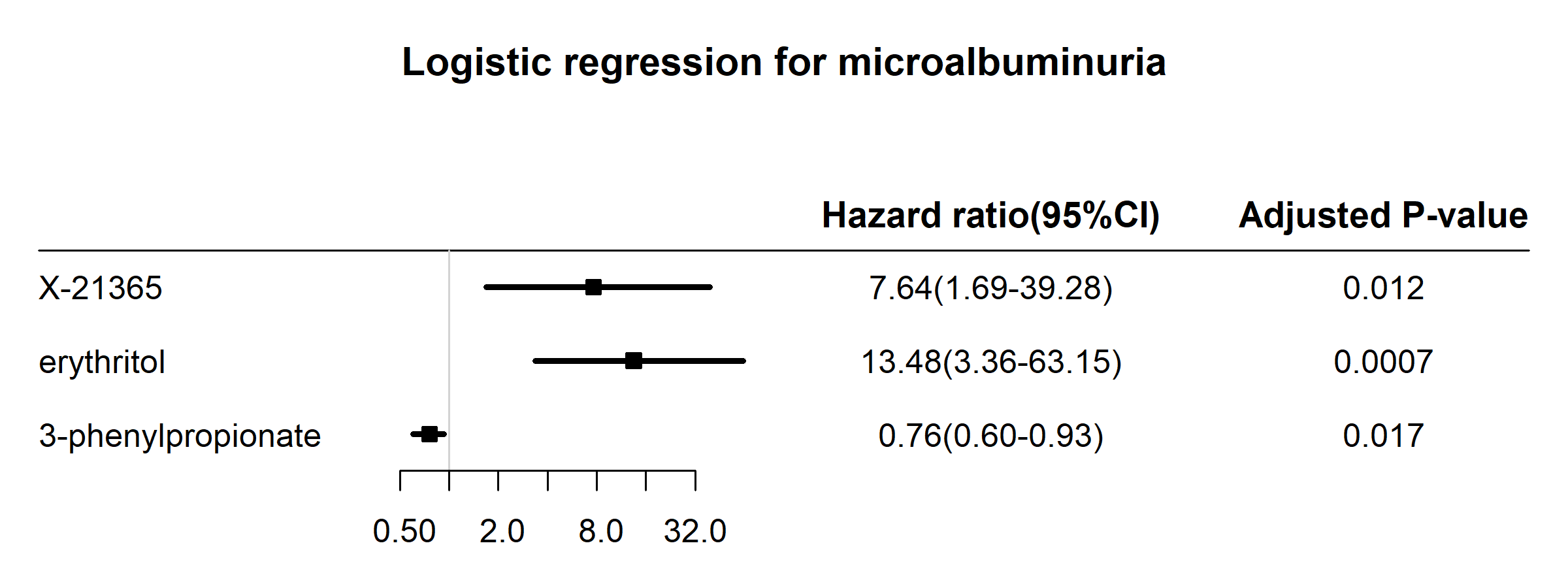


# Supplementary Figure 4 — ROC-curve

Prediction of incident microalbuminuria when the three top metabolites (N-trimethyl-5-aminovalerate, erythritol and 3-phenylpropionate, red line) added on top of the clinical model including age of diabetes onset, baseline AER and long-term mean HbA_1c_ (metabolites only blue line). The three metabolites significantly improved also when long-term HbA_1c_ was included in the clinical model (ROC_AUC_=0.82 *vs* 0.72).


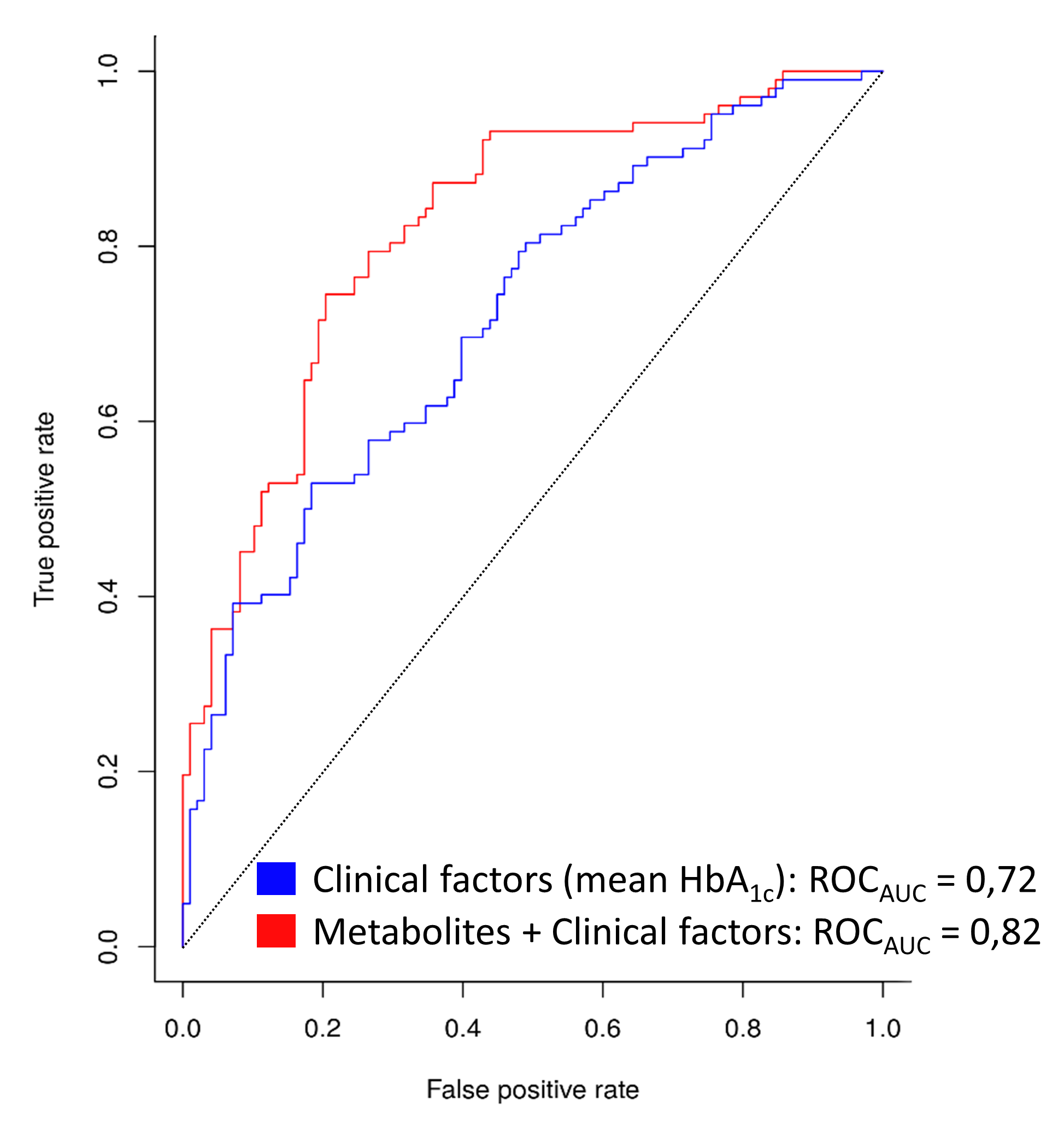


Supplementary Table 1 – Metabolomic profile of selected metabolites.*

|  | **Progressors** | **Non-progressors** | ***p*** |
| --- | --- | --- | --- |
| **Carbohydrates** |  |  |  |
| Erythritol | 1.03 ± 0.29 | 0.95 ± 0.27 | <0.0001 |
| Glucose | 1.11 ± 0.46 | 0.99 ± 0.42 | ns |
| Sorbitol | 1.09 ± 0.71 | 0.79 ± 0.60 | 0.0013 |
| Lactate | 1.07 ± 0.25 | 0.98 ± 0.25 | 0.0090 |
| 1,5-anhydroglucitol | 1.19 ± 1.35 | 1.69 ± 1.54 | 0.0047 |
| Succinate (TCA cycle) | 1.06 ± 0.23 | 0.99 ± 0.15 | 0.0138 |
| Malate (TCA cycle) | 1.13 ± 0.41 | 1.00 ± 0.35 | 0.0195 |
| **Aminoacids (AA)** |  |  |  |
| Alanine | 1.05 ± 0.24 | 0.95 ± 0.24 | 0.0499 |
| Glutamine | *1.13 ± 1.03* | *1.37 ± 0.99* | *0.0034* |
| X-21365 | *1.07 ± 0.29* | *0.93 ± 0.20* | <0.0001 |
| Glutarate (lysine metabolism) | 1.16 ± 0.43 | 0.99 ± 0.42 | 0.0009 |
| 3-phenylpropionate (phenylalanine & tyrosine) | 1.23 ± 1.65 | 2.05 ± 2.47 | *0.0001* |
| C-glycosyltryptophan (tryptophan) | 1.07 ± 0.21 | 0.99 ± 0.16 | 0.0123 |
| 2-hydroxybutyrate (methionine and cysteine) | 1.32 ± 0.70 | 1.05 ± 0.57 | 0.0018 |
| 5-oxoproline (glutathione metabolism) | 0.96 ± 0.31 | 1.09 ± 0.29 | *0.0021* |
| **γ-glutamyl AA and dipeptides** |  |  |  |
| γ-glutamylglutamate | 0.90 ± 0.45 | 1.13 ± 0.42 | *<0.0001* |
| cis-Cyclo[L-ala-L-Pro] | 1.09 ± 0.36 | 0.97 ± 0.25 | 0.0048 |
| **Fatty acids (FA)** |  |  |  |
| Valerate (short-chain) | 1.21 ± 0.64 | 1.02 ± 0.54 | 0.0122 |
| 22:2n6 (PUFA) | 1.29 ± 0.71 | 1.11 ± 0.63 | 0.042 |
| Heptanedioate (dicarboxylic fatty acids) | 1.16 ± 0.47 | 1.00 ± 0.36 | 0.006 |
| Hydroxybutyrylcarnitine (acylcarnitines) | 1.74 ± 1.86 | 1.21 ± 1.67 | 0.0048 |
| Carnitine | 1.04 ± 0.16 | 0.98 ± 0.13 | 0.0011 |
| α-hydroxycaproate (monohydroxy FA) | 1.10 ± 0.34 | 0.99 ± 0.40 | 0.0087 |
| Leukotriene B4 | 1.10 ± 0.33 | 0.97 ± 0.28 | 0.0028 |
| 1-stearoylglycerophosphoethanolamine | 1.22 ± 0.49 | 1.02 ± 0.39 | 0.001 |
| Glycerol | 1.14 ± 0.42 | 0.94 ± 0.35 | 0.0034 |
| 1-stearoylglycerol | 1.15 ± 0.42 | 0.98 ± 0.29 | 0.001 |
| 1,3-dipalmitoylglycerol | 1.14 ± 0.99 | 0.87 ± 0.82 | 0.0428 |
| **Steroids** |  |  |  |
| Cortisone | 1.04 ± 0.26 | 0.93 ± 0.21 | 0.0024 |
| **Bile acids** |  |  |  |
| Chenodeoxycholate | 1.24 ± 1.43 | 1.89 ± 3.55 | *0.0417* |
| Deoxycholate | 1.35 ± 1.23 | 1.09 ± 0.88 | 0.0189 |
| Deoxycholate/chenodeoxycholate | 2.27 ± 5.21 | 1.31 ± 3.09 | 0.0113 |
| **Nucleotides** |  |  |  |
| N6-carbamoylthreonyladenosine (purines) | 1.06 ± 0.21 | 0.99 ± 0.167 | 0.0197 |
| Pseudouridine (pyrimidine) | 1.05 ± 0.18 | 0.99 ± 0.13 | 0.0072 |
| N3-methyluridine (pyrimidine) | 1.04 ± 0.22 | 0.97 ± 0.18 | 0.0241 |

* data are in R.I. (relative intensity), scaled to a median of 1, and summarized as mean ± SD

*p* values are from Welch test. Lower values in progressors are in italics.

# Supplementary Table 2 – 111 metabolites with nominally significant P-value after adjustment for clinical factors

| **BIOCHEMICAL** | **Sub-Pathway** | **Platform** | **Non-Progressors** | **Progressors** | **adj *p* value** |
| --- | --- | --- | --- | --- | --- |
| **AMINO ACID** | |  |  |  |  |
| N-acetylalanine | Alanine and Aspartate Metabolism | LC/MS neg | 0.9941 | 1.0304 | 0.007144 |
| glutarate (pentanedioate) | Lysine Metabolism | LC/MS pos | 0.9895 | 1.1613 | 0.023758 |
| pipecolate | Lysine Metabolism | LC/MS pos | 1.0397 | 1.2332 | 0.027351 |
| methionine sulfone | Methionine. Cysteine. SAM and Taurine Metabolism | LC/MS pos | 1.0146 | 1.077 | 0.010029 |
| N-acetylmethionine sulfoxide | Methionine. Cysteine. SAM and Taurine Metabolism | LC/MS neg | 0.9684 | 1.0357 | 0.008049 |
| 3-phenylpropionate (hydrocinnamate) | Phenylalanine and Tyrosine Metabolism | LC/MS neg | 2.0514 | 1.2335 | 0.01312 |
| N-acetylputrescine | Polyamine Metabolism | LC/MS pos | 1.0198 | 1.1769 | 0.007317 |
| C-glycosyltryptophan* | Tryptophan Metabolism | LC/MS pos | 0.9974 | 1.0675 | 0.005386 |
| pro-hydroxy-pro | Urea cycle; Arginine and Proline Metabolism | LC/MS pos | 0.9737 | 1.187 | 0.002185 |
| **CARBOHYDRATE** | |  |  |  |  |
| erythritol | Food Component/Plant | GC/MS | 0.9474 | 1.1118 | 0.000493 |
| mannitol | Fructose. Mannose and Galactose Metabolism | GC/MS | 1.6349 | 2.7165 | 0.005734 |
| sorbitol | Fructose. Mannose and Galactose Metabolism | GC/MS | 0.795 | 1.0943 | 0.006198 |
| arabinose | Pentose Metabolism | GC/MS | 0.9344 | 1.114 | 0.033305 |
| xylonate | Pentose Metabolism | GC/MS | 0.9514 | 1.0815 | 0.035812 |
| **COFACTORS AND VITAMINS** | | |  |  |  |
| nicotinate | Nicotinate and Nicotinamide Metabolism | LC/MS neg | 0.9426 | 1.1051 | 0.045332 |
| gamma-tocopherol | Tocopherol Metabolism | GC/MS | 0.4521 | 0.6424 | 0.035519 |
| **ENERGY** |  |  |  |  |  |
| malate | TCA Cycle | GC/MS | 1.0016 | 1.1291 | 0.036131 |
| succinate | TCA Cycle | LC/MS neg | 0.9926 | 1.0637 | 0.036408 |
| **LIPID** |  |  |  |  |  |
| carnitine | Carnitine Metabolism | LC/MS pos | 0.975 | 1.0447 | 0.046253 |
| 2-aminoheptanoate | Fatty Acid. Amino | LC/MS pos | 1.0352 | 1.1787 | 0.014965 |
| docosadioate | Fatty Acid. Dicarboxylate | LC/MS neg | 1.495 | 1.2226 | 0.012391 |
| pimelate (heptanedioate) | Fatty Acid. Dicarboxylate | LC/MS pos | 1.0021 | 1.1644 | 0.049877 |
| suberate (octanedioate) | Fatty Acid. Dicarboxylate | LC/MS neg | 1.0552 | 1.1878 | 0.043594 |
| octanoylcarnitine | Fatty Acid Metabolism(Acyl Carnitine) | LC/MS pos | 1.1112 | 1.4933 | 0.040628 |
| propionylglycine | Fatty Acid Metabolism (also BCAA Metabolism) | LC/MS neg | 1.2348 | 1.0615 | 0.003095 |
| glycerol | Glycerolipid Metabolism | GC/MS | 0.985 | 1.1436 | 0.018131 |
| pentadecanoate (15:0) | Long Chain Fatty Acid | GC/MS | 1.007 | 1.1289 | 0.02323 |
| 1-docosahexaenoylglycerophosphocholine (22:6n3)* | Lysolipid | LC/MS pos | 1.2654 | 1.079 | 0.025073 |
| 1-docosapentaenoylglycerophosphocholine (22:5n3)* | Lysolipid | LC/MS pos | 1.132 | 1.0362 | 0.036602 |
| 1-palmitoylglycerophosphoethanolamine | Lysolipid | LC/MS neg | 1.0514 | 1.263 | 0.020279 |
| 2-docosahexaenoylglycerophosphocholine* | Lysolipid | LC/MS pos | 1.2643 | 1.038 | 0.017646 |
| 2-palmitoylglycerophosphoethanolamine* | Lysolipid | LC/MS neg | 1.0451 | 1.2471 | 0.032868 |
| 1-stearoylglycerol (1-monostearin) | Monoacylglycerol | GC/MS | 0.9805 | 1.1457 | 0.004776 |
| docosatrienoate (22:3n3) | Polyunsaturated Fatty Acid (n3 and n6) | LC/MS neg | 1.0852 | 1.3168 | 0.030027 |
| glycoursodeoxycholate | Secondary Bile Acid Metabolism | LC/MS neg | 1.7304 | 1.3478 | 0.048664 |
| taurocholenate sulfate | Secondary Bile Acid Metabolism | LC/MS neg | 1.1623 | 1.2243 | 0.01554 |
| 3b-hydroxy-5-cholenoic acid | Secondary Bile Acid Metabolism | LC/MS neg | 1.3214 | 0.9975 | 0.030048 |
| corticosterone | Steroid | LC/MS pos | 1.4399 | 1.2097 | 0.043722 |
| cortisone | Steroid | LC/MS neg | 0.9347 | 1.0447 | 0.001522 |
| testosterone | Steroid | LC/MS pos | 1.1423 | 0.9554 | 0.027567 |
| 7-hydroxycholesterol (alpha or beta) | Sterol | LC/MS pos | 1.2663 | 1.1241 | 0.026685 |
| **NUCLEOTIDE** | |  |  |  |  |
| methylphosphate | Purine and Pyrimidine Metabolism | GC/MS | 0.9552 | 1.0954 | 0.007444 |
| N6-carbamoylthreonyladenosine | Purine Metabolism. Adenine containing | LC/MS pos | 0.9934 | 1.0584 | 0.022091 |
| N2.N2-dimethylguanosine | Purine Metabolism. Guanine containing | LC/MS pos | 0.9897 | 1.0481 | 0.049113 |
| 2'-deoxyguanosine | Purine Metabolism. Guanine containing | LC/MS neg | 0.9484 | 1.7899 | 0.042342 |
| urate | Purine Metabolism. (Hypo)Xanthine/Inosine containing | LC/MS neg | 0.9608 | 1.0785 | 0.010196 |
| N3-methyluridine | Pyrimidine Metabolism. Uracil containing | LC/MS pos | 0.971 | 1.041 | 0.017475 |
| pseudouridine | Pyrimidine Metabolism. Uracil containing | LC/MS pos | 0.9908 | 1.0545 | 0.002776 |
| **PEPTIDE** |  |  |  |  |  |
| arginylleucine | Dipeptide | LC/MS pos | 1.3892 | 2.4896 | 0.016124 |
| cis-Cyclo[L-ala-L-Pro] | Dipeptide | LC/MS pos | 0.97 | 1.0928 | 0.012096 |
| cyclo(L-phe-L-pro) | Dipeptide | LC/MS pos | 1.1274 | 1.5127 | 0.006554 |
| glycylproline | Dipeptide | LC/MS pos | 1.0942 | 1.2567 | 0.044403 |
| tryptophylasparagine | Dipeptide | LC/MS pos | 1.2709 | 1.6691 | 0.033135 |
| gamma-glutamylglutamate | Gamma-glutamyl Amino Acid | LC/MS neg | 1.1345 | 0.8991 | 0.006585 |
| gamma-glutamylleucine | Gamma-glutamyl Amino Acid | LC/MS pos | 1.1296 | 0.9868 | 0.041815 |
| gamma-glutamyllysine | Gamma-glutamyl Amino Acid | LC/MS pos | 1.3292 | 1.0383 | 0.039934 |
| glu-glu-glu | Polypeptide | LC/MS neg | 1.3044 | 0.9393 | 0.035392 |
| **XENOBIOTICS** | |  |  |  |  |
| 3-methyl catechol sulfate (2) | Benzoate Metabolism | LC/MS neg | 1.5642 | 1.2904 | 0.040309 |
| O-sulfo-L-tyrosine | Chemical | LC/MS neg | 1.0185 | 1.0904 | 0.031632 |
| cinnamoylglycine | Food Component/Plant | LC/MS neg | 2.1665 | 1.4607 | 0.025591 |
| ferulic acid 4-sulfate | Food Component/Plant | LC/MS neg | 1.4805 | 1.2096 | 0.047277 |
| **UNKNOWN** |  |  |  |  |  |
| X - 11360 | N/A | LC/MS pos | 1.1011 | 1.4801 | 0.012545 |
| X - 11429 | N/A | LC/MS neg | 1.0073 | 1.0608 | 0.005181 |
| X - 12026 | N/A | LC/MS neg | 0.9797 | 1.0775 | 0.049714 |
| X - 12027 | N/A | LC/MS neg | 1.0113 | 1.0811 | 0.042206 |
| X - 12039 | N/A | LC/MS neg | 1.8378 | 1.3231 | 0.037976 |
| X - 12096 | N/A | LC/MS pos | 0.9743 | 1.1552 | 0.029343 |
| X - 12117 | N/A | LC/MS pos | 0.803 | 1.2235 | 0.001457 |
| X - 12329 | N/A | LC/MS neg | 2.5682 | 1.7187 | 0.043111 |
| X - 12408 | N/A | LC/MS neg | 1.6945 | 1.1728 | 0.00252 |
| X - 12690 | N/A | LC/MS pos | 0.9759 | 1.0661 | 0.001638 |
| X - 12718 | N/A | LC/MS neg | 0.7019 | 1.1095 | 0.034309 |
| X - 12798 | N/A | LC/MS pos | 0.9692 | 1.119 | 0.048441 |
| X - 12833 | N/A | LC/MS neg | 1.7812 | 1.322 | 0.022668 |
| X - 12844 | N/A | LC/MS neg | 1.012 | 1.1752 | 0.0133 |
| X - 13772 | N/A | LC/MS neg | 1.0582 | 1.3186 | 0.029341 |
| X - 14374 | N/A | LC/MS pos | 0.9766 | 1.1689 | 0.000851 |
| X - 14658 | N/A | LC/MS neg | 1.4863 | 1.5702 | 0.018563 |
| X - 15728 | N/A | LC/MS neg | 1.8524 | 1.3578 | 0.020314 |
| X - 16124 | N/A | LC/MS neg | 47.4623 | 14.3575 | 0.008314 |
| X - 16564 | N/A | LC/MS neg | 1.4756 | 1.2322 | 0.030661 |
| X - 17010 | N/A | LC/MS neg | 0.9588 | 1.0792 | 0.015687 |
| X - 17138 | N/A | LC/MS pos | 0.996 | 1.1977 | 0.049191 |
| X - 17145 | N/A | LC/MS neg | 1.5541 | 1.0224 | 0.003291 |
| X - 17269 | N/A | LC/MS neg | 0.9744 | 1.0878 | 0.02495 |
| X - 17320 | N/A | LC/MS pos | 1.9226 | 1.5147 | 0.046141 |
| X - 17339 | N/A | LC/MS pos | 0.9734 | 1.1648 | 0.007036 |
| X - 17346 | N/A | LC/MS pos | 1.4109 | 1.1315 | 0.028457 |
| X - 17349 | N/A | LC/MS neg | 1.1366 | 0.7867 | 0.049837 |
| X - 17357 | N/A | LC/MS neg | 0.9506 | 1.2134 | 0.000971 |
| X - 17359 | N/A | LC/MS neg | 0.9629 | 1.1205 | 0.002482 |
| X - 17367 | N/A | LC/MS neg | 1.6759 | 1.4384 | 0.04394 |
| X - 17690 | N/A | LC/MS neg | 2.01 | 3.2975 | 0.03384 |
| X - 17842 | N/A | LC/MS neg | 0.9122 | 1.148 | 0.007051 |
| X - 17969 | N/A | LC/MS neg | 1.0886 | 1.2743 | 0.034952 |
| X - 18750 | N/A | LC/MS neg | 0.3966 | 1.7267 | 0.037204 |
| X - 19220 | N/A | LC/MS neg | 1.5909 | 2.7993 | 0.020326 |
| X - 20529 | N/A | LC/MS neg | 1.5427 | 0.9957 | 0.030303 |
| X - 20587 | N/A | LC/MS pos | 1.0515 | 1.2682 | 0.025223 |
| X - 20731 | N/A | LC/MS neg | 0.944 | 1.124 | 0.011241 |
| X - 21365 | N/A | LC/MS pos | 0.9613 | 1.1118 | 0.011075 |
| X - 21441 | N/A | LC/MS neg | 1.1089 | 1.3557 | 0.01916 |
| X - 21442 | N/A | LC/MS neg | 1.6262 | 1.1016 | 0.042783 |
| X - 21471 | N/A | LC/MS neg | 1.0278 | 1.1544 | 0.018907 |
| X - 21583 | N/A | LC/MS neg | 0.9951 | 1.0002 | 0.038359 |
| X - 21736 | N/A | LC/MS neg | 1.0302 | 1.4029 | 0.041954 |
| X - 21756 | N/A | LC/MS pos | 0.9921 | 1.0615 | 0.00136 |
| X - 22099 | N/A | LC/MS neg | 1.0669 | 1.4602 | 0.030327 |
| X - 22379 | N/A | LC/MS neg | 1.2383 | 1.0282 | 0.03981 |
| X - 22475 | N/A | LC/MS pos | 1.0794 | 1.3762 | 0.020186 |
| X - 22518 | N/A | LC/MS pos | 1.0021 | 1.2135 | 0.049057 |

* indicates compounds that have not been officially confirmed based on a standard, but Metabolon Inc. are confident in their identity

*p*-values adjusted for age of diabetes onset, baseline HbA_1c_ and AER

# Supplementary Table 3 – The Finnish Diabetic Nephropathy Study Centers

Anjalankoski Health Center S.Koivula, T.Uggeldahl

Central Finland Central Hospital, Jyväskylä T.Forslund, A.Halonen, A.Koistinen, P.Koskiaho,

M.Laukkanen, J.Saltevo, M.Tiihonen

Central Hospital of Åland Islands, Mariehamn M.Forsen, H.Granlund, A.-C.Jonsson, B.Nyroos

Central Hospital of Kanta-Häme, Hämeenlinna P.Kinnunen, A.Orvola, T.Salonen, A.Vähänen

Central Hospital of Kymenlaakso, Kotka R.Paldanius, M.Riihelä, L.Ryysy

Central Hospital of Länsi-Pohja, Kemi H.Laukkanen, P.Nyländen, A.Sademies

Central Ostrobothnian Hospital District, Kokkola S.Anderson, B.Asplund, U.Byskata, P.Liedes,

M.Kuusela, T.Virkkala

City of Espoo Health Center:

Espoonlahti A.Nikkola, E.Ritola

Tapiola M.Niska, H.Saarinen

Samaria E.Oukko-Ruponen, T.Virtanen

Viherlaakso A.Lyytinen

City of Helsinki Health Center:

Puistola H.Kari, T.Simonen

Suutarila A.Kaprio, J.Kärkkäinen, B.Rantaeskola

Töölö P.Kääriäinen, J.Haaga, A-L.Pietiläinen

City of Hyvinkää Health Center S.Klemetti, T.Nyandoto, E.Rontu, S.Satuli-Autere

City of Vantaa Health Center:

Korso R.Toivonen, H.Virtanen

Länsimäki R.Ahonen, M.Ivaska-Suomela, A.Jauhiainen

Martinlaakso M.Laine, T.Pellonpää, R.Puranen

Myyrmäki A.Airas, J.Laakso, K.Rautavaara

Rekola M.Erola, E.Jatkola

Tikkurila R.Lönnblad, A.Malm, J.Mäkelä, E.Rautamo

Heinola Health Center P.Hentunen, J.Lagerstam

Helsinki University Central Hospital, Department of

Medicine, Division of Nephrology A.Ahola, M.Feodoroff, D.Gordin, O.Heikkilä, K.Hietala, M.Korolainen J.Kytö, S.Lindh, K.Pettersson-Fernholm, A.Sandelin, L.Thorn, J.Tuomikangas, T.Vesisenaho, J.Wadén

Herttoniemi Hospital, Helsinki V.Sipilä

Hospital of Lounais-Häme, Forssa T.Kalliomäki, J.Koskelainen, R.Nikkanen,

N.Savolainen, H.Sulonen, E.Valtonen

Hyvinkää Hospital L. Norvio, A. Hämäläinen

Iisalmi Hospital E.Toivanen

Jokilaakso Hospital, Jämsä A.Parta, I.Pirttiniemi

Jorvi Hospital, Helsinki University Central Hospital S.Aranko, S.Ervasti, R.Kauppinen-Mäkelin,

A.Kuusisto, T.Leppälä, K.Nikkilä, L.Pekkonen

Jyväskylä Health Center, Kyllö K.Nuorva, M.Tiihonen

Kainuu Central Hospital, Kajaani S.Jokelainen, K.Kananen, M.Karjalainen, P.Kemppainen, A-M.Mankinen, A.Reponen

M.Sankari

Kerava Health Center H.Stuckey, P.Suominen

Kirkkonummi Health Center A.Lappalainen, M.Liimatainen, J.Santaholma

Kivelä Hospital, Helsinki A.Aimolahti, E.Huovinen

Koskela Hospital, Helsinki V.Ilkka, M.Lehtimäki

Kotka Health Center E.Pälikkö-Kontinen, A.Vanhanen

Kouvola Health Center E.Koskinen, T.Siitonen

Kuopio University Hospital E.Huttunen, R.Ikäheimo, P.Karhapää, P.Kekäläinen,

M.Laakso, T.Lakka, E.Lampainen, L.Moilanen,

L.Niskanen, U.Tuovinen, I.Vauhkonen, E.Voutilainen

Kuusamo Health Center T.Kääriäinen, E.Isopoussu

Kuusankoski Hospital E.Kilkki, I.Koskinen, L.Riihelä

Laakso Hospital, Helsinki T.Meriläinen, P.Poukka, R.Savolainen, N.Uhlenius

Lahti City Hospital A.Mäkelä, M.Tanner

Lapland Central Hospital, Rovaniemi L.Hyvärinen, K.Lampela, S.Pöykkö, T.Rompasaari, S.Severinkangas, T.Tulokas

Lappeenranta Health Center P. Erola, L. Härkönen, P.Linkola, I.Pulli, E.Repo

Lohja Hospital T.Granlund, K.Hietanen, M.Porrassalmi, M.Saari, T.Salonen, M.Tiikkainen,

Länsi-Uusimaa Hospital, Tammisaari I.-M.Jousmaa, J.Rinne

Loimaa Health Center A.Mäkelä, P.Eloranta

Malmi Hospital, Helsinki H.Lanki, S.Moilanen, M.Tilly-Kiesi

Mikkeli Central Hospital A.Gynther, R.Manninen, P.Nironen, M.Salminen,

T.Vänttinen

Mänttä Regional Hospital I.Pirttiniemi, A-M.Hänninen

North Karelian Hospital, Joensuu U-M.Henttula, P.Kekäläinen, M.Pietarinen,

A.Rissanen, M.Voutilainen

Nurmijärvi Health Center A.Burgos, K.Urtamo

Oulaskangas Hospital, Oulainen E.Jokelainen, P-L.Jylkkä, E.Kaarlela, J.Vuolaspuro

Oulu Health Center L.Hiltunen, R.Häkkinen, S.Keinänen-Kiukaanniemi

Oulu University Hospital R.Ikäheimo

Päijät-Häme Central Hospital H.Haapamäki, A.Helanterä, S.Hämäläinen,

V.Ilvesmäki, H.Miettinen

Palokka Health Center P.Sopanen, L.Welling

Pieksämäki Hospital V.Sevtsenko, M.Tamminen

Pietarsaari Hospital M-L.Holmbäck, B.Isomaa, L.Sarelin

Pori City Hospital P.Ahonen, P.Merisalo, E.Muurinen, K.Sävelä

Porvoo Hospital M.Kallio, B.Rask, S.Rämö

Raahe Hospital A.Holma, M.Honkala, A.Tuomivaara, R.Vainionpää

Rauma Hospital K.Laine, K.Saarinen, T.Salminen

Riihimäki Hospital P.Aalto, E.Immonen, L.Juurinen

Salo Hospital A.Alanko, J.Lapinleimu, P.Rautio, M.Virtanen

Satakunta Central Hospital, Pori M.Asola, M.Juhola, P.Kunelius, M.-L.Lahdenmäki,

P.Pääkkönen, M.Rautavirta

Savonlinna Central Hospital T.Pulli, P.Sallinen, M.Taskinen, E.Tolvanen, T.Tuominen

H.Valtonen, A.Vartia, S-L.Viitanen

Seinäjoki Central Hospital O.Antila, E.Korpi-Hyövälti, T.Latvala, E.Leijala, T.Leikkari, M.Punkari N.Rantamäki, H.Vähävuori

South Karelia Central Hospital, Lappeenranta T.Ensala, E.Hussi, R.Härkönen, U.Nyholm, J.Toivanen

Tampere Health Center A.Vaden, P.Alarotu, E.Kujansuu, H.Kirkkopelto-Jokinen,

M.Helin, S.Gummerus, L.Calonius, T.Niskanen, T.Kaitala,

T.Vatanen

Tampere University Hospital I.Ala-Houhala, R.Kannisto, T.Kuningas, P.Lampinen, M.Määttä,

H.Oksala, T.Oksanen, A.Putila, H.Saha, K.Salonen, H.Tauriainen,

S.Tulokas

Tiirismaa Health Center, Hollola T.Kivelä, L.Petlin, L.Savolainen

Turku Health Center A.Artukka, I.Hämäläinen, L.Lehtinen, E.Pyysalo, H.Virtamo, M.Viinikkala, M.Vähätalo

Turku University Central Hospital K.Breitholz, R.Eskola, K.Metsärinne, U.Pietilä,

P.Saarinen, R.Tuominen, S.Äyräpää

Vaajakoski Health Center K.Mäkinen, P.Sopanen

Valkeakoski Regional Hospital S.Ojanen, E.Valtonen, H.Ylönen, M.Rautiainen,

T.Immonen

Vammala Regional Hospital I.Isomäki, R.Kroneld, L.Mustaniemi, M.Tapiolinna-Mäkelä

Vasa Central Hospital S.Bergkulla, U.Hautamäki, V-A.Myllyniemi, I.Rusk
